# Bioactive metabolites from the leaves of *Withania adpressa*

**DOI:** 10.1080/13880209.2018.1499781

**Published:** 2018-11-17

**Authors:** Widad Ben Bakrim, Laila El Bouzidi, Jean-Marc Nuzillard, Sylvian Cretton, Noémie Saraux, Aymeric Monteillier, Philippe Christen, Muriel Cuendet, Khalid Bekkouche

**Affiliations:** a Department of Biology, Faculty of Sciences Semlalia, Laboratory of Biotechnology, Protection and Valorisation of Plant Resources (URAC35 association Unit), Cadi Ayyad University, Marrakech, Morocco;; b Institut de Chimie Moléculaire de Reims, UMR CNRS 7312, SFR Cap-Santé FED 4231, UFR de Pharmacie, Université de Reims Champagne-Ardenne, Reims, France;; c School of Pharmaceutical Sciences, University of Geneva, University of Lausanne, Geneva 4, Switzerland

**Keywords:** Withanolides, wadpressine, antioxidant activity, NF-κB inhibition, antiproliferative activity

## Abstract

**Context:***Withania* (Solanaceae) species are known to be a rich source of withanolides, which have shown several biological properties.

**Objective:** To identify the compounds responsible for *Withania adpressa* Coss. antioxidant activity and further test them for their NF-κB inhibition and antiproliferative activity in multiple myeloma cells.

**Materials and methods**: Compounds were obtained from the EtOAc extract of *W. adpressa* leaves. Structure elucidation was carried out mainly by 1D- and 2D-NMR, and mass spectrometry. Isolated compounds were tested in a dose-response for their *in vitro* NF-κB inhibition and antiproliferative activity in multiple myeloma cells after 5 and 72 h treatment, respectively.

**Results:** The fractionation resulted in the isolation of a new glycowithanolide named wadpressine (**5**) together with withanolide F, withaferin A, coagulin L, and nicotiflorin. The latter showed a moderate ability to scavenge free radicals in DPPH (IC_50_ = 35.3 µM) and NO (IC_50_ = 41.3 µM) assays. Withanolide F and withaferin A exhibited low µM antiproliferative activity against both multiple myeloma cancer stem cells and RPMI 8226 cells. Furthermore, they inhibited NF-κB activity with IC_50_ values of 1.2 and 0.047 µM, respectively. The other compounds showed a moderate inhibition of cell proliferation in RPMI 8226 cells, but were inactive against cancer stem cells and did not inhibit NF-κB activity.

**Discussion and conclusions:** One new glycowithanolide and four known compounds were isolated. Biological evaluation data gave further insight on the antitumor potential of withanolides for refractory cancers.

## Introduction

The genus *Withania* (Solanaceae) consists of eight species (The Plant List 2013) occurring predominantly in North Africa and from the Mediterranean basin to India (Hepper 1991). This genus is known for elaborating withanolides, which are steroidal lactones characterized by a C28 basic skeleton. Since the isolation of withaferin A (Lavie et al. 1965), more than 750 withanolides with various functional groups have been isolated, largely but not exclusively, from about 25 genera of Solanaceae (Hang et al. 2012). In recent years, withanolides have attracted a significant attention from numerous researchers owing to their structural features and their multiple bioactivities such as cytotoxic (Cordero et al. 2009; Hang et al. 2012), anti-inflammatory (Jayaprakasam and Nair 2003), immunomodulatory (Mesaik et al. 2006), anticholinesterase (Choudhary et al. 2005), and antioxidant (Bhattacharya et al. 2001) properties.

The flora of Morocco includes three *Withania* species: *W. frutescens* Pauquy*, W. somnifera* (L.) Dunal, and *W. adpressa* Coss. The latter is a medicinal plant endemic to Moroccan Sahara, locally known as “aglim” or “hjuju”, and used to treat food intoxication (Bellakhdar 1997). Our previous phytochemical study on this species led to the isolation of a new withanolide named (22*R*)-14α,15α,17β,20β-tetrahydroxy-1-oxowitha-2,5,24-trien-26,22-olide together with withanolides F and J (Abdeljebbar et al. 2007). To date, there is only one pharmacological study on *W. adpressa*, which mainly refers to the potent cytotoxicity of its withanolides against human cancer cells (Abdeljebbar et al. 2009).

The crude extracts of *W. somnifera* and *W. frutescens* were reported to have *in vitro* and *in vivo* antioxidant activities (Bhattacharya et al. 2001; El Bouzidi et al. 2011). The compounds responsible for the antioxidant activity included glycowithanolides (Bhattacharya et al. 1997). Interest has increased in naturally occurring antioxidants since they may be used to protect humans from oxidative stress damage and to lower the incidence of various cancers. Furthermore, *Withania* spp. extracts and several withanolides isolated from them have shown potential for their antitumor activity (Mathur et al. 2006).

**Figure 1.**
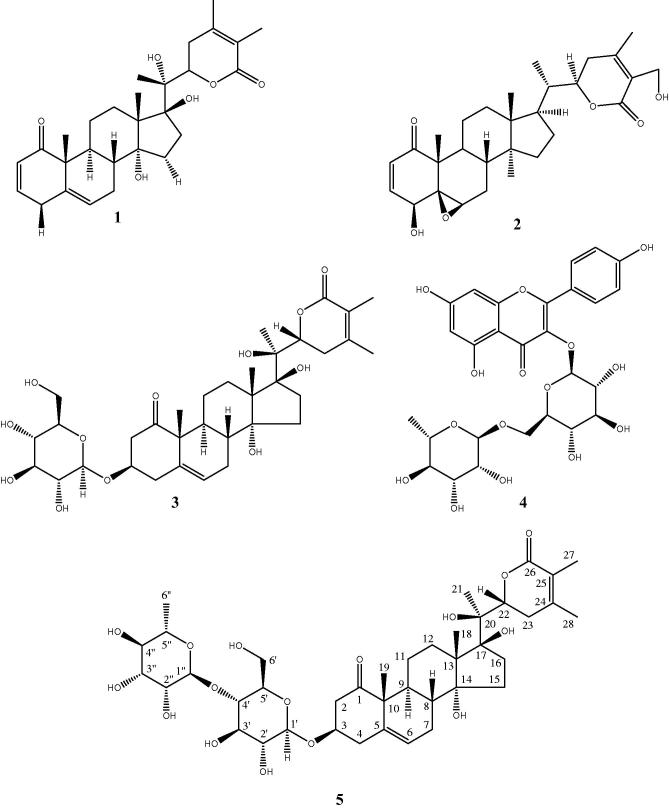
Chemical structures of compounds isolated from *W. adpressa* leaves.

To investigate the potential of *W. adpressa* leaves for cancer chemoprevention and therapy, the EtOAc extract was selected for bioactivity-guided fractionation based on its antioxidant activity. Four known compounds (**1–4**, Figure 1) and a new glycowithanolide, named wadpressine (**5**), were isolated. To further investigate their potential to control carcinogenesis, pure compounds were tested for their ability to inhibit NF-κB, a transcription factor involved in various aspects of the carcinogenesis process such as inflammation, cell survival, proliferation, migration, and angiogenesis (Aggarwal 2004). Finally, these compounds were tested against two multiple myeloma (MM) cell lines. MM is a hematological cancer in which the over activation of the NF-κB pathway is particularly crucial (Demchenko and Kuehl 2010).

## Materials and methods

### General experimental procedures

UHPLC was performed on an Ultimate 3000 UPLC System (ThermoFisher Scientific, Waltham, MA) with an Acquity BEH C_18_ column (50 × 2.1 mm i.d.; 1.7 μm, Waters, Milford, MA) using an optimized gradient (MeCN and H_2_O both containing 0.1% formic acid) of 5–98% MeCN in 4 min and followed by a washing step with 98% MeCN for 2 min. HRMS spectra were obtained on a Q Exactive Plus Hybrid quadripole-orbitrap mass spectrometer (ThermoFisher Scientific) using electrospray in positive and negative modes. The spray voltage was set at 4.0 and 2.5 kV, and the sheath gas flow rate (N_2_) at 47.5 and 50 units, respectively, the capillary temperature at 255 °C; the S lens RF level at 50 and the probe heater temperature at 412.5 °C. ^1^H- and ^13^C-NMR spectra were recorded on a Varian Unity Inova 500 MHz NMR (Palo Alto, CA) instrument. Chemical shifts are reported in parts per million (*δ*) using the residual CD_3_OD signal (*δ*_H_ 3.31; *δ*_C_ 49.0) or the CDCl_3_ signal (*δ*_H_ 7.26; *δ*_C_ 77.2) as internal standards for ^1 ^H- and ^13 ^C-NMR, respectively, and coupling constants (*J*) are reported in Hz. Complete assignment was performed based on 2 D experiments (COSY, ROESY, NOESY, edited-HSQC and HMBC).

Silica gel 60 (230–400 mesh, Fluka Analytical, Darmstadt, Germany), Sephadex LH-20 (Pharmacia Biotech, Uppsala, Sweden) and Lichroprep RP-18 (40–63 μm, Merck, Darmstadt, Germany) were used for column chromatography. Fractions were monitored by thin layer chromatography (TLC) on precoated silica gel 60F_254_ (0.20 mm film thickness, Merck). Spots were visualized by exposure to UV light and/or by spraying with Godin or sulphuric vanillin reagents followed by heating at 105 °C.

### Plant material

The leaves of *W. adpressa* were collected at the flowering stage in Taznaght (South of Morocco) in April 2015 and authenticated by Dr Aziz Abbad, Department of Biology, Cadi Ayyad University. A voucher specimen (N° Mar 4223) was deposited at the Botany Department Herbarium, Faculty of Sciences-Semlalia, Cadi Ayyad University, Marrakech, Morocco.

### Extraction

Air-dried powdered leaves of *W. adpressa* (2 kg) were defatted with hexane and then extracted with methanol using a Soxhlet apparatus for 72 h for each solvent. After removing the solvent under reduced pressure, the methanol extract (168 g) was suspended into distilled water, filtered and then partitioned successively with dichloromethane, ethyl acetate, and *n*-butanol to obtain 28, 15, and 48 g of crude extracts, respectively.

### Biological assays

#### 2,2-Diphenyl-1-picrylhydrazyl (DPPH) radical scavenging assay

The DPPH radical scavenging activity was carried out as described previously (Şahin et al. 2004) with slight modifications. Samples (1–200 µg/mL) were added to a 60-μM methanol solution of DPPH. The absorbance of the solution was measured at 517 nm after a 30-min incubation period at room temperature in the dark.

#### NF-κB inhibition assay

NF-κB inhibitory activity was assessed using a HEK293/NF-κB-luc cell line (Panomics, Fremont, CA) as previously described (Ndongo et al. 2017). Briefly, cells were cultured at 37 °C and 5% CO_2_ atmosphere in high glucose Dulbecco's modified Eagle's medium (ThermoFisher Scientific) with 100 IU/mL penicillin, 100 μg/mL streptomycin, 100 μg/mL hygromycin B (ThermoFisher Scientific) and 10% foetal bovine serum (Biowest, Nuaillé, France). The day before the assay, cells were treated for 1 h with 2.5 μM of Cell Tracker Green CMFDA (ThermoFisher Scientific), a fluorescent dye used to quantify cell viability, in FBS-free medium. Then, 10^4^ cells/well were seeded in 96-well plates and incubated overnight. After that, cells were treated with either the vehicle control (0.5% DMSO in culture medium) or the samples and stimulated for 5 h with 20 ng/mL of TNF-α (Sigma-Aldrich, Saint Louis, MO). Finally, cells were lysed with reporter lysis buffer (Promega, Madison, WI) and fluorescence signals were read on a Cytation 3 multimode plate reader (Biotek, Winooski, VT). Luciferase assay reagent (Promega) was then added to each well using the auto-injector and the luminescence signals were read. The fluorescence signal was used to normalize the luminescence signal for each well to limit the influence of cell toxicity in luminescence signal intensity. Relative NF-κB activity was quantified by comparing the normalized luminescence signal of vehicle treated nonstimulated cells with the one of vehicle treated stimulated cells. All pure compounds were first screened at 20 μM. Compounds able to inhibit more than 50% of TNF-α induced NF-κB activity at this concentration were considered as active and were selected for calculation of their IC_50_. Nonlinear regression (with sigmoidal dose response) was used to calculate the IC_50_ values using GraphPad Prism. Each compound was tested in duplicate and three independent experiments were performed using parthenolide as positive control.

#### Antiproliferative activity

Human multiple myeloma cancer stem cells (MM-CSCs) derived from the bone marrow of a MM patient, were obtained from Celprogen (Torrance, CA). MM-CSCs were used between passages 4 and 12 for experiments. The tumour plasma cells RPMI 8226 were obtained from LGC standards (Middlesex, United Kingdom). RPMI 8226 cells were used between passages 10 and 25 for experiments. The two cell lines were cultured at 37 °C and 5% CO_2_ atmosphere in RPMI 1640 culture medium supplemented with 10% foetal bovine serum (Biowest), 100 IU/mL penicillin and 250 μg/mL streptomycin. MTT and XTT assays were used to evaluate the antiproliferative activity of the pure compounds in MM-CSCs and RPMI 8226 cells, respectively (Issa and Cuendet 2017). MM-CSCs were seeded in 96 well plates at a density of 5,000 cells per well and allowed to adhere for 24 h, whereas RPMI 8226 cells were plated at a density of 15,000 cells per well and treated immediately. MM-CSCs and RPMI 8226 cells were treated with increasing concentrations (0–20 µM) of compounds for 72 h. Twenty microlitres of MTT solution (5 mg/mL) or 50 μL XTT solution (1 mg/mL) were added in each well and incubated for 2 h (MTT assay) or 4 h (XTT assay). The media and MTT solution were aspirated and the cells containing formazan were solubilized in 100 µL DMSO. Absorbance was measured at 590 nm (MTT assay) or 450 nm (XTT assay). The percentage of cell viability was calculated as the absorbance of each well was divided by that of the vehicle control wells (0.5% DMSO in culture medium) and multiplied by 100. IC_50_ values were calculated using GraphPad Prism. Each compound was tested in triplicate and three independent experiments were performed using bortezomib as positive control.

## Results and discussion

### Compound isolation and characterization

Three extracts of increasing polarity (CH_2_Cl_2_, EtOAc, and *n*-BuOH) were prepared from the leaves of *W. adpressa* and tested for their antioxidant properties. The EtOAc extract exhibited the highest radical scavenging activity (IC_50_ = 4.8 µg/mL). Based on this result, this extract (15 g) was fractionated by low pressure chromatography on a silica gel column (100 × 2.5 cm i.d.) and eluted with a gradient made of CH_2_Cl_2_–EtOAc–MeOH (95:5:1 to 0:10:90). Fractions were combined based on their TLC profile to afford 13 fractions (F1–F13). Fractions F3, F6, F7, and F8 showed the highest DPPH free radical scavenging activity with IC_50_ values ranging between 10.5 and 25.4 µg/mL. Fraction F3 (783 mg) was further separated on a RP-18 column with a H_2_O–MeOH mixture of increasing polarity (75:25 to 25:75). The active subfractions F3-2 (230 mg) and F3-3 (133 mg) were subjected to a final purification on a Sephadex LH-20 column with MeOH as eluent to yield compounds **1** (50 mg) and **2** (25 mg), respectively. Fraction F6 (1.4 g) was further fractionated on a silica gel column with a CH_2_Cl_2_-MeOH mixture of increasing polarity (9:1 to 2:8) to give six subfractions. Subfraction F6-6 (600 mg) was submitted to a final purification on a Sephadex LH-20 eluted with MeOH to yield compound **3** (150 mg). Fraction F7 was subjected to column chromatography on a RP-18 column and eluted with a gradient of H_2_O-MeOH (75:25 to 25:75) and afforded six subfractions. The active subfraction F7-5 was submitted to purification on a Sephadex LH-20 column with MeOH to give compound **4** (25 mg). Fraction F8 (520 mg) was separated on Sephadex LH-20 with MeOH and afforded five subfractions. Subfraction F8-4 was submitted to column chromatography on a RP-18 column and eluted with H_2_O–MeOH (75:25 to 25:75). Final purification was performed by preparative TLC using CH_2_Cl_2_–EtOAC–MeOH (8:2:0.1) to afford compound **5** (34 mg).

Compounds **1–4** were identified as withanolide F (Abdeljebbar et al. 2007), withaferin A (Lavie et al. 1965; Erazo et al. 2008), coagulin L (Atta-ur-Rahman et al. 1998), and nicotiflorin (De Sousa et al. 2014), respectively. Their structure elucidation was performed by high resolution mass spectrometry, 1D- and 2D-NMR experiments and comparison with spectroscopic data reported in the literature. These compounds are described for the first time in this species except for withanolide F (**1**), which was previously isolated from the leaves of this species (Abdeljebbar et al. 2007). Withaferin A (**2**) was previously isolated from *W. somnifera*, *W. coagulans*, *W. aristata*, and *W. frutescens* (Gonzalez et al. 1982; Neogi et al. 1988; Llanos et al. 2010) and coagulin L (**3**) from *W. coagulans* (Atta-ur-Rahman et al. 1998). It is noteworthy that this is the first report on the isolation of nicotiflorin (**4**) from a *Withania* species.

Compound **5** was isolated as a white amorphous powder. HRESIMS showed a pseudo molecular ion peak at *m/z* 797.3853 [M + H]^+^ (calcd. 797.3864), indicating a molecular formula of C_40_H_60_O_16_. IR spectrum revealed the presence of a hydroxyl (3,420 cm^−1^), an α,β-unsaturated δ-lactone moiety (1715 cm^−1^) and a carboxyl (1690 cm^−1^). ^1 ^H- and ^13 ^C-NMR data are given in Table 1. The structure of compound **5** was partly deduced from that of coagulin L (**3**). The comparison of their ^13 ^C-NMR chemical shifts (Table S1) showed that **5** contains an additional sugar unit in position 4′ compared to **3** (8 ppm deviation). The planar structure of compound **3** was determined by the analysis of ^1 ^H, ^13 ^C, COSY, HSQC and HMBC spectra and found to be identical to the one of coagulin L (Atta-ur-Rahman et al. 1998). The comparison of ^13^C-NMR chemical shifts of 2,3-dihydro-3β-hydroxywithanolide F (the aglycone of coagulin L) (Zhang and Timmermann 2016) or of the aglycone part of tetra-acetylated coagulin L (Atta-ur-Rahman et al. 1998), with those of compound **3**, let us to conclude the identity of the latter was coagulin L, even though chemical shift comparison was biased by the use of different NMR solvents (CDCl_3_ and CD_3_OD) (Table 1). The absence of reported stereoisomers of coagulin L provides further support for our conclusion. The sugar unit in **5** is bound to the aglycone in position 3, as shown by the H-1′/C-3 correlation. A series of COSY correlations, starting from H-1′, revealed the chemical shifts of H-2′ to H-5′, all of these being in axial position, as deduced from the high value of their coupling constants. The HSQC spectrum indicated the chemical shift of H-6′a and H-6′b as those of a methylene group in a primary alcohol functional group whose connection with C-5′ was proven by the HMBC spectrum. The anomeric configuration of this glucose unit is β, as indicated by the high H-1′/H-2′ coupling constant (7.8 Hz).

**Table 1.** ^1^H-(500 MHz) and ^13^C-(125 MHz) NMR data of compound **5** (CDCl_3_).

					COSY	HMBC	ROESY
Position	*δ*(^13^C)	*δ*(^1^H)	^a^	^1^H multiplicity	H->H	H->C	H->H
1	214.3						
2	46.97	2.74	2H	d, 6.9	3	1, 3, 4	3, 9(w), 19, 1′
3	77.17	3.98		m	2, 4a, 4b	1	2, 4a, 1′
4	38.96	2.69	a, eq, α	dd, 13.7, 6.3	3, 4b	2, 3, 5, 6, 10	3, 4b, 6, 1′
		2.49	b, ax, β	m	3, 4a	5(w)	4a, 19, 1′ (w)
5	136.63						
6	126.95	5.69		bs	7b	10	4a, 7a, 7b
7	27.05	2.10	a	m	7b	5(w), 6(w)	6, 7b, 15b(w)
		1.90	b	m	6, 7a		6, 7a, 15b
8	37.22	1.92		m	9		9, 15a, 18, 19
9	37.22	2.14		m	8, 11a		2(w), 11a, 11b(w), 12a
10	54.46						
11	23.31	1.65	a, eq, αb,	m	9, 11b, 12a		9, 12a
		1.60	ax, β	m	9, 11a, 12a		9(w), 12a(w), 18
12	31.6	2.35	a, ax, α	dt, 12, 5	11a, 11b, 12b		9, 11a, 11b(w), 12b, 22
		1.30	b, eq, β	m	12a		12a, 18, 22
13	55.88						
14	84.1						
15	33.31	1.74	a, β	m	15b, 16a		8, 15b, 18
		1.51	b, α	dd, 12.3, 8.7	15a, 16a	13, 14, 17	7a(w), 7b, 15a, 16a
16	37.61	2.57	a, α	m	15a, 15b, 16b		15b, 16b, 21
		1.57	b, β	m	16a	14, 17	16a, 19, 21
17	88.94						
18	21.13	1.12	3H	s		12, 13, 14, 17	8, 11b, 12b, 15a
19	18.74	1.29	3H	s		1, 5, 9, 10	2, 4b, 8, 16b
20	80.01						
21	19.58	1.38	3H	s		17, 20, 22	16a, 16b
22	83.11	4.84		m	23a, 23b		12a, 12b, 23a, 23b(w)
23		2.65	a, eq	dd, 18.8, 2.6	22, 23b	24, 35	22, 23b, 28
	35.85	2.52	b, ax	m	22, 23a, 27		22(w), 23a
24	153.52						
25	122.12						
26	169.25						
27	12.47	1.85	3H	s	23b	24, 25, 26	28
28	20.68	1.96	3H	s		23, 24, 25, 26(w)	23a, 27
1′	103.16	4.39		d, 7.8	2′	3	2, 3, 4a, 4b(w), 2′(w), 3′, 5′
2′	75.32	3.18		dd, 8.1, 9	1′, 3′		1′(w), 4′
3′	76.87	3.44		t, 9	2′, 4′	2′, 4′	1′, 1″(w)
4′	79.71	3.52		t, 9.1	3′, 5′	5′, 6′	2′, 6′a, 6′b, 1″
5′	76.93	3.33		m	4′, 6′a		1′, 6′a, 6′b
6′	62.06	3.80	a	dd, 12.3, 1.2	5′, 6′b	4′, 5′	4′, 5′, 6′b, 1″(w)
		3.65	b	dd, 12.3, 4.2	6′a		4′, 5′, 6′a, 1″(w)
1″	103.06	4.84		bs		4′, 3″, 5″	3′ (w), 4′, 6′a(w), 6′b(w), 2″
2″	72.6	3.83		bs		3″, 4″	1″, 3″
3″	72.35	3.62		dd, 9.7, 3.4	4″	4″	5″, 2″
4″	73.9	3.40		t, 9.5	3″, 5″	3″, 5″(w), 6″	
5″	70.81	3.96		m	4″, 6″		3″, 6″
6″	17.98	1.26	3H	d, 6.9	6″	4″, 5″	5″

a Number of H atoms, 1 if not given. H names a and b are assigned so that *δ*(Ha) > *δ*(Hb).

Chemical shifts using *δ*(CD_3_OD) = 49.15 and *δ*(CD_2_HOD) = 3.31.

The anomeric position 1″ of the sugar unit present in **5** was characterized by ^1 ^H and ^13 ^C chemical shifts clearly identified in the HSQC spectrum. The H-1″ signal, hidden under the strong HOD signal of the solvent, correlated with the one of H-2″ in the TOCSY spectrum. The COSY spectrum sequentially correlated with the signal of H-2″ to those of H-3″ to H-6″. The latter appeared as a shielded doublet of a methyl group. The small value of the H-1″/H-2″ and H-2″/H-3″ coupling constants and the high value of the H-3″/H-4″ and H-4″/H-5″ coupling constants are compatible with a rhamnose unit in α configuration. The H-1″/C-4′ HMBC correlation confirmed the binding of this sugar to position 4′ of the glucose unit. Therefore, the structure of compound **5** was determined as (14*R*,17*S*,20*R*,22*R*)-3β-*O*-(α-l-rhamnopyranosyl-(1→4)-β-d-glucopyranosyl)-14,17,20-trihydroxy-1-oxo-witha-5,24-dienolide and named wadpressine. The common characteristic of coagulin L (**3**) and wadpressine (**5**) is the presence of a 5-en-1-one structure that had only been reported in withanolides from *Withania coagulans* (Atta-ur-Rahman et al. 1998) and *Ajuga parviflora* Benth. (Lamiaceae) (Khan et al. 1999).

### NF-κB inhibitory activity

More recently, it has become clear that an over activation of the NF-κB pathway plays a critical role in cancer development and progression (Aggarwal and Sung 2011). Hence, each compound was also tested for its NF-κB inhibition. Only withanolide F (**1**) and withaferin A (**2**), an already described NF-κB inhibitor (Heyninck et al. 2014), inhibited TNF-α-induced NF-κB activity with IC_50_ values of 1.2 and 0.05 μM, respectively (Table 2).

**Table 2.** NF-ĸB inhibition mediated by the isolated compounds (**1**-**5**)^a^.

Compounds	IC_50_ [µM] ± SD
**1**	1.2 ± 0.2
**2**	0.05 ± 0.01
**3**	> 20
**4**	> 20
**5**	> 20
Parthenolide^b^	0.53 ± 0.01

a IC_50_ values represent the means ± standard deviation of three independent measurements.

b Positive control.

### Antiproliferative activity

MM remains an incurable malignancy, with a median survival of only 5 years, despite all the treatment advances (Fonseca et al. 2017). The presence of CSCs in MM is considered to contribute to disease relapse through their drug-resistant nature. Targeting MM-CSCs is therefore a promising strategy for MM treatment. The effect of pure compounds on cell growth in MM-CSCs and RPMI 8226 cells was investigated using MTT and XTT assays (Table 3). Withaferin A (**2**) exhibited the strongest activity with IC_50_ values of 0.33 and 0.17 µM in MM-CSCs and RPMI 8226 cells, respectively. Similar results were reported for withaferin A in a previous study (Issa and Cuendet 2017).

**Table 3.** Antiproliferative activity of the isolated compounds (**1**–**5**) in MM-CSCs and RPMI 8226 cells^a^.

	IC_50_ [µM] ± SD		
Compounds	MM-CSCs	RPMI 8226	
**1**	5.3 ± 0.9	0.1 ± 0.01	
**2**	0.33 ± 0.02	0.17 ± 0.03	
**3**	>20	3.6 ± 0.6	
**4**	>20	7.1 ± 0.1	
**5**	>20	3.2 ± 0.4	
Bortezomib^b^	0.0091 ± 0.0014	0.0033 ± 0.0008	

a IC_50_ values represent the means ± standard deviation of three measurements.

b Positive control.

Withanolide F (**1**) also showed activity against both cell lines (Table 3). Previous results have shown that withanolide F strongly inhibited the growth of several cancer cell lines (Jayaprakasam et al. 2003; Abdeljebbar et al. 2009). Compounds **3–5** were only active against RPMI 8226 cells. Interestingly, the two compounds able to inhibit NF-κB activity were also the ones active on MM-CSCs, highlighting the potential interest of NF-κB inhibitors for the treatment of refractory cancers.

## Conclusions

One new glycowithanolide (**5**) and four known compounds (**1–4**) were identified from the leaves of *W. adpressa*. Compounds (**2**–**4**) were isolated from this plant for the first time. In addition, results from the biological evaluation of the compounds isolated from *W. adpressa* leaves highlight the interest of this plant for its antitumor properties.

## Supplementary Material

Supplementary Material
