# Supplementary material for: Bioactive metabolites from the leaves of Withania adpressa
Source: Pharm Biol. 2018 Nov 17;56(1):505–10. doi: 10.1080/13880209.2018.1499781 (PMC6249549; doi:10.1080/13880209.2018.1499781)
Supplement: Supplementary Material [file IPHB_A_1499781_SM1130.docx]

**SUPPLEMENTARY DATA**

**Bioactive metabolites from the leaves of *Withania adpressa***

Widad Ben Bakrim^a^, Laila El Bouzidi^a^, Jean-Marc Nuzillard^b^, Sylvian Cretton^c^, Noémie Saraux^c^, Aymeric Monteillier^c^, Philippe Christen^c^, Muriel Cuendet^c^, Khalid Bekkouche^a^

^a^ *Laboratory of Biotechnology, Protection and Valorisation of Plant Resources (URAC35 association Unit), Cadi Ayyad University, Department of Biology, Faculty of Sciences Semlalia, POB 2390, Marrakech, Morocco*

^b^ *Institut de Chimie Moléculaire de Reims, UMR CNRS 7312, SFR Cap-Santé FED 4231, UFR de Pharmacie, Université de Reims Champagne-Ardenne, 51687 Reims CEDEX 2, France*

^c^ *School of Pharmaceutical Sciences, University of Geneva, University of Lausanne, 1211 Geneva 4, Switzerland*

ORCID numbers:

Philippe Christen 0000-0003-4713-0323

Muriel Cuendet 0000-0002-1523-1907

Corresponding author: Muriel Cuendet, *E-mail address*: [muriel.cuendet@unige.ch](mailto:muriel.cuendet@unige.ch)

**Table S1**

^13^C NMR comparison of compounds **3**, **5**, aglycone of coagulin L (2,3-dihydro-3*β*-hydroxywithanolide F) and aglycone of tetra-acetylated coagulin L (CDCl_3_)

| Position | **3** | **5** | Aglycone of coagulin L[23] | Aglycone of acetylated coagulin L [28] |
| --- | --- | --- | --- | --- |
| 1 | 214.37 | 214.30 | 209.7 | 210.3 |
| 2 | 46.94 | 46.97 | 47.6 | 46 |
| 3 | 76.98 | 77.17 | 68.6 | 77 |
| 4 | 38.94 | 38.96 | 40 | 38.2 |
| 5 | 136.65 | 136.63 | 135.4 | 133.9 |
| 6 | 126.92 | 126.95 | 125.9 | 126.8 |
| 7 | 27.05 | 27.05 | 25.9 | 25.8 |
| 8 | 37.23 | 37.22 | 36.2 | 36.1 |
| 9 | 37.23 | 37.22 | 35.9 | 35.5 |
| 10 | 54.46 | 54.46 | 53.1 | 54.6 |
| 11 | 23.30 | 23.31 | 22.2 | 21.9 |
| 12 | 31.60 | 31.60 | 30.4 | 30 |
| 13 | 55.88 | 55.88 | 54.1 | 52.7 |
| 14 | 84.10 | 84.10 | 82.5 | 81.8 |
| 15 | 33.30 | 33.31 | 34.6 | 32.3 |
| 16 | 37.62 | 37.61 | 37.1 | 37.5 |
| 17 | 88.93 | 88.94 | 87.9 | 87.9 |
| 18 | 19.59 | 19.58 | 20.6 | 20.2 |
| 19 | 18.73 | 18.74 | 18.4 | 18.7 |
| 20 | 80.00 | 80.01 | 78.7 | 78.9 |
| 21 | 21.13 | 21.13 | 19.1 | 19.5 |
| 22 | 83.12 | 83.11 | 81.5 | 80.3 |
| 23 | 35.84 | 35.85 | 32.5 | 34.2 |
| 24 | 153.52 | 153.52 | 152.3 | 150.8 |
| 25 | 122.12 | 122.12 | 121.4 | 121.3 |
| 26 | 169.25 | 169.25 | 166 | 166.3 |
| 27 | 12.47 | 12.47 | 12.4 | 12.2 |
| 28 | 20.68 | 20.68 | 20.7 | 20.3 |
| 1' | 103.16 | 103.16 |  |  |
| 2' | 75.18 | 75.32 |  |  |
| 3' | 78.17 | 76.87 |  |  |
| 4' | 71.71 | 79.71 |  |  |
| 5' | 78.08 | 76.93 |  |  |
| 6' | 62.88 | 62.06 |  |  |
| 1" |  | 103.06 |  |  |
| 2" |  | 72.60 |  |  |
| 3" |  | 72.35 |  |  |
| 4" |  | 73.90 |  |  |
| 5" |  | 70.81 |  |  |
| 6" |  | 17.98 |  |  |

**NMR data**

Wadpressine 1D and 2D NMR raw data and spectra of wadpressine (compound **5**) are stored in an archive file named wadpressine.zip in Bruker format. This file can be temporarily accessed from <https://www.dropbox.com/s/y93hr2zrgxyi65e/Wadpressine.zip?dl=0>. and later permanently from <https://doi.org/10.5281/zenodo.1233301>. NMR files can be opened with the TopSpin software, which is free for academics.
